# Smoking in children's playgrounds: Can we do more to protect our children?

**DOI:** 10.18332/tpc/182911

**Published:** 2024-02-05

**Authors:** José Precioso, Regina F. Alves, Catarina Samorinha

**Affiliations:** 1 Research Centre on Child Studies (CIEC), Institute of Education, University of Minho, Braga, Portugal; 2 Research Institute for Medical and Health Sciences, University of Sharjah, Sharjah, United Arab Emirates

**Keywords:** secondhand smoke exposure, child, playground, smoking prevention, smoke-free policy

**Dear Editor,**

Smoking continues to be the leading cause of preventable death and disease worldwide^1^. The exposure of children to tobacco smoke can have a decisive influence on their future behavior, with a high probability that they will become smokers^2,3^. On 1 January 2008, the Law on smoking control came into force in Portugal, prohibiting the sale of tobacco to minors and banning smoking in all public areas, including outdoor areas of education establishments^4^. Ten years later, Law No. 63/2017 of 3 August came into force, which aims to protect children from smoking and declares a ban on smoking in the settings intended explicitly for children, namely: ‘... kindergartens, nurseries, and other childcare facilities, children and youth homes, leisure centers, summer camps and camps, playgrounds, and other similar facilities’^5^. Additionally, Law No. 88/2019 of 3 September^6^ in Portugal approves measures for the collection and treatment of tobacco waste, and punishes those who throw cigarette butts on public roads with fines of 25–250 €. This study aimed to describe children's exposure to tobacco smoke in playgrounds and to assess compliance with the above laws.

This observational study took place in playgrounds in Northern Portugal in October and November 2022. It was inspired by the project ‘Tackling secondhand exposure to tobacco smoke and aerosols of electronic cigarettes: the TackSHS project protocol’ (TackSHS project)^7^. The convenience sample consisted of 35 children’s playgrounds located in 16 cities. At least five people had to be present in the playground for the observations to be made. The main variables observed were the presence of tobacco smoke smell, the number of people smoking inside and outside the playground (traditional cigarettes, electronic cigarettes, or heated tobacco), the number of cigarette butts on the ground, and the presence of non-smoking signs. Approximately 15 minutes were spent by the researcher observing each playground and collecting data inside and outside the place (<1 m around the playground). Results from this pilot study showed that the smell of tobacco smoke was present in 3 out of 35 playgrounds; the proportion of people smoking inside the playground was 2 out of 35, and 7 out of 35 people were smoking inside. The proportion of people smoking e-cigarettes and heated cigarettes (inside or outside the playground) was 5 out of 35. Regarding cigarette butts, 7/35 and 30/35 playgrounds had cigarette butts inside and outside, respectively, and three playgrounds were littered with heated cigarette butts (inside and outside). Only two of the 35 playgrounds observed had signs indicating that smoking was prohibited.

This study showed that many children may still be exposed to smoking behavior in playgrounds, not only because smokers were detected during our assessment but also because of the indirect evidence of the high number of cigarette butts observed on the ground. Apart from evidence of cigarette smoking, this study showed signs of using e-cigarettes and heated tobacco products in observed places. This study has demonstrated an apparent lack of compliance with measures related to the collection and treatment of tobacco waste, such as throwing cigarette butts on public roads. Placing non-smoking signs and educational posters next to playgrounds is highly recommended, as the law requires. In addition, awareness campaigns are required to raise consciousness about the need to dispose of cigarette butts in the proper trash bins, and to implement health education measures to promote smoking prevention and cessation, thereby reducing the number of children exposed to tobacco smoke.

## Data Availability

Data sharing is not applicable to this article as no new data were created.
